# Light/Dark Shifting Promotes Alcohol-Induced Colon Carcinogenesis: Possible Role of Intestinal Inflammatory Milieu and Microbiota

**DOI:** 10.3390/ijms17122017

**Published:** 2016-12-02

**Authors:** Faraz Bishehsari, Abdulrahman Saadalla, Khashayarsha Khazaie, Phillip A. Engen, Robin M. Voigt, Brandon B. Shetuni, Christopher Forsyth, Maliha Shaikh, Martha Hotz Vitaterna, Fred Turek, Ali Keshavarzian

**Affiliations:** 1 Department of Medicine, Division of Gastroenterology, Rush University Medical Center, Chicago, IL 60612, USA; Phillip_Engen@rush.edu (P.A.E.); Robin_Voigt@rush.edu (R.M.V.); Christopher_Forsyth@rush.edu (C.F.); Maliha_Shaikh@rush.edu (M.S.); Ali_Keshavarzian@rush.edu (A.K.); 2 Department of Immunology, Mayo Clinic College of Medicine, Mayo Clinic, Rochester, MN 55905, USA; Saadalla.Abdulrahman@mayo.edu (A.S.); Khazaie@mayo.edu (K.K.); 3 Northwestern Medicine, Central DuPage Hospital, Winfield, IL 60190, USA; Brandon.Shetuni@CadenceHealth.org; 4 Center for Sleep and Circadian Biology, Northwestern University, Evanston, IL 60208, USA; m-vitaterna@northwestern.edu (M.H.V.); fturek@northwestern.edu (F.T.)

**Keywords:** colon cancer, alcohol, circadian disruption, inflammation, microbiota

## Abstract

Background: Colorectal cancer (CRC) is associated with the modern lifestyle. Chronic alcohol consumption—a frequent habit of majority of modern societies—increases the risk of CRC. Our group showed that chronic alcohol consumption increases polyposis in a mouse mode of CRC. Here we assess the effect of circadian disruption—another modern life style habit—in promoting alcohol-associated CRC. Method: TS4Cre × adenomatous polyposis coli (APC)^lox468^ mice underwent (a) an alcohol-containing diet while maintained on a normal 12 h light:12 h dark cycle; or (b) an alcohol-containing diet in conjunction with circadian disruption by once-weekly 12 h phase reversals of the light:dark (LD) cycle. Mice were sacrificed after eight weeks of full alcohol and/or LD shift to collect intestine samples. Tumor number, size, and histologic grades were compared between animal groups. Mast cell protease 2 (MCP2) and 6 (MCP6) histology score were analyzed and compared. Stool collected at baseline and after four weeks of experimental manipulations was used for microbiota analysis. Results: The combination of alcohol and LD shifting accelerated intestinal polyposis, with a significant increase in polyp size, and caused advanced neoplasia. Consistent with a pathogenic role of stromal tryptase-positive mast cells in colon carcinogenesis, the ratio of mMCP6 (stromal)/mMCP2 (intraepithelial) mast cells increased upon LD shifting. Baseline microbiota was similar between groups, and experimental manipulations resulted in a significant difference in the microbiota composition between groups. Conclusions: Circadian disruption by Light:dark shifting exacerbates alcohol-induced polyposis and CRC. Effect of circadian disruption could, at least partly, be mediated by promoting a pro-tumorigenic inflammatory milieu via changes in microbiota.

## 1. Introduction

Colorectal cancer (CRC) is the second leading cause of cancer-associated mortality in the US [1]. Only a small portion of colorectal cancers are caused by known genetic syndromes, while most CRC cases are sporadic, without a strong familial background [2]. Immigration and epidemiological studies provide compelling evidence of an association between CRC incidence and the modern lifestyle [1,3]. This is further confirmed by studies showing a rapid rise of CRC risk in immigrants from low-risk areas who immigrated to Western/high-risk countries [3,4]. Despite the established link of CRC with the overall phenomenon of “Westernization”, this knowledge has not yet been translated to our approach in risk stratification and preventive strategies for CRC [5]. There are only weak associations between each individual environmental factor and the disease risk [6]; thus, it is highly likely that additive or synergistic effects from a combination of risk factors have a large impact on CRC susceptibility [6,7]. Identifying factors and mechanisms that mediate life-style impact on CRC could help us to better stratify our population for CRC screening and design novel therapeutic approaches.

Chronic alcohol consumption—a frequent habit of modern societies—is a known risk factor for CRC, as shown in several population-based studies [8,9]. Only subsets of individuals who drink alcohol are at risk for CRC, suggesting that there may be other environmental or genetic co-factor(s) that predispose individuals to alcohol-induced colon carcinogenesis. A better understanding of the mechanisms that mediate alcohol-induced effects on intestinal tumorigenesis could help us to identify such co-factors.

Alcohol consumption causes intestinal inflammation, which is associated with accelerated polyposis [10,11]. We have shown that intestinal inflammation from alcohol is exacerbated by the disruption of circadian rhythms from shifting light:dark (LD) cycles [12,13,14]. This is not surprising, as up to a third of the genome—including a variety of cellular processes and immune regulatory mechanisms—are under circadian control [15,16]. The central circadian clock in the suprachiasmatic nucleus (SCN) is entrained by the LD cycle; thus, alterations in the LD cycle leads to the disruption of circadian rhythms [17]. The ensuing circadian disruption is associated with the disruption of tissue homeostasis, chronic inflammatory status, and increased susceptibility to cancer in general [18,19,20,21]. In fact, shift work—resulting in circadian rhythm disruption—increases the risk of some malignancies (including CRC) in some epidemiological studies [22,23].

Here, we hypothesized that circadian rhythm disruption could promote the alcohol-induced effects on colon carcinogenesis. We assessed the effect of LD shift—a frequent habit of our 24/7 society—in combination with alcohol consumption in an animal model of CRC. Consistent with the known effect of circadian rhythm disruption on immunity, we found that LD shift increased intestinal tumorigenesis in alcohol-fed mice by promoting a pro-tumorigenic inflammatory milieu.

## 2. Results/Discussion

### 2.1. Light:Dark (LD) Shift Enhances Alcohol-Induced Colon Cancer Carcinogenesis

**Figure 1:**
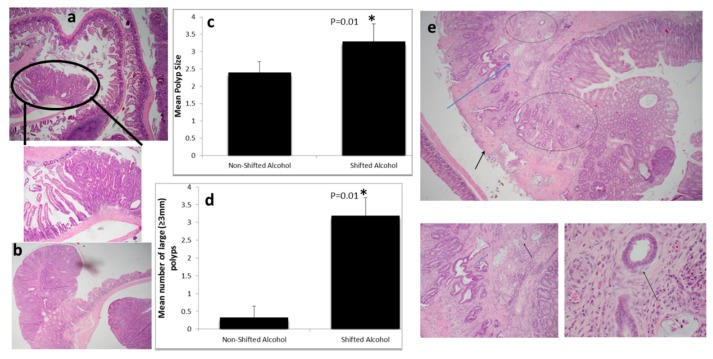
Light:dark (LD) shift accelerates intestinal tumorigenesis in alcohol treated mice: (**a**) Tubular adenoma in the ileum (4×). The circle is a 10× view of the same lesion, showing clear delineation between adenoma (**right**) and adjacent normal mucosa (**left**); (**b**) Colonic tubular adenoma (4×); (**c**) Polyp size (* *p* = 0.01) and (**d**) number of large polyps (* *p* = 0.01) were increased significantly by light:dark shifting; (**e**) Only mice in the experimental group developed invasive colon cancer; picture shows invasive adenocarcinoma emerging from a tubular adenoma. Two glands immediately above muscularis mucosa (MM) (MM is indicated by the blue arrow) are infiltrating the mucosa, and a few glands in top center have infiltrated the submucosa (top circle). Area of invasion to MM and submucosa is also observed in the center-bottom (blue circle). Part of the polyp surface was eroded—an inconsequential finding (black arrow). Right-bottom panel is a larger view (10×) of the same polyp, showing glands infiltrating into the MM (blue arrow) and beyond (black arrow). The left-bottom panel is the high power view (40×) of two glands that have infiltrated into the submucosa. At this power, desmoplasia is apparent as a subtle rim around the glands (black arrow); desmoplasia is an unequivocal feature of invasive carcinomas. Note: 4×, 10×, and 40× fields are 25.0 microns, 10.0 microns, and 2.5 microns in diameter, respectively.

All (five of five) LD shifted alcohol-fed mice (experimental group) developed advanced neoplasia; three had lesions with carcinoma in situ, and two with submucosa invasion. The experimental group had a greater number of polyps (9.2 ± 1.5 vs. 6.6 ± 2.6) and lesions with carcinoma in situ (4.4 ± 0.9 vs. 2.6 ± 1.5) than the non-shifted alcohol-fed mice, although these differences did not reach statistical significance due to limited sample sizes. Polyps were significantly larger in the experimental group (mean diameter of 3.3 ± 0.5 vs. 2.2 ± 0.3 mm, *p* = 0.01). Furthermore, there was a significant increase in the number of large (≥3.0 mm) polyps in the experimental group (3.2 ± 0.5 vs. 0.3 ± 0.3, *p* = 0.01) (Figure 1).

Most importantly, all alcohol-fed shifted mice had at least two lesions with histopathologic features of advanced adenoma (carcinoma in situ or more advanced), compared to only 1/3 of the control group (5/5, 100% vs. 1/3, 33%, *p* = 0.03).

Invasive colon cancer was present only in alcohol-fed shifted mice. Two mice in the experimental group developed invasive CRC, while none of the alcohol-fed mice without LD shift developed CRC (Figure 1). Interestingly, both cases of invasive cancer had minimal high grade dysplasia, suggesting that there could have been high grade dysplasia that was overrun by the invasive cancer, or alternatively, epithelial cells acquired invasive capacity rather quickly in response to LD shift and alcohol together, causing a rapid transition from adenoma to invasive cancer. Overall, these findings are consistent with enhanced colon carcinogenesis in response to a combination of circadian disruption and alcohol versus alcohol alone.

### 2.2. Circadian Disruption along with Alcohol Feeding Results in a Shift from Intraepithelial MCP2^+^ to Stromal MCP6^+^ Mast Cells

**Figure 2:**
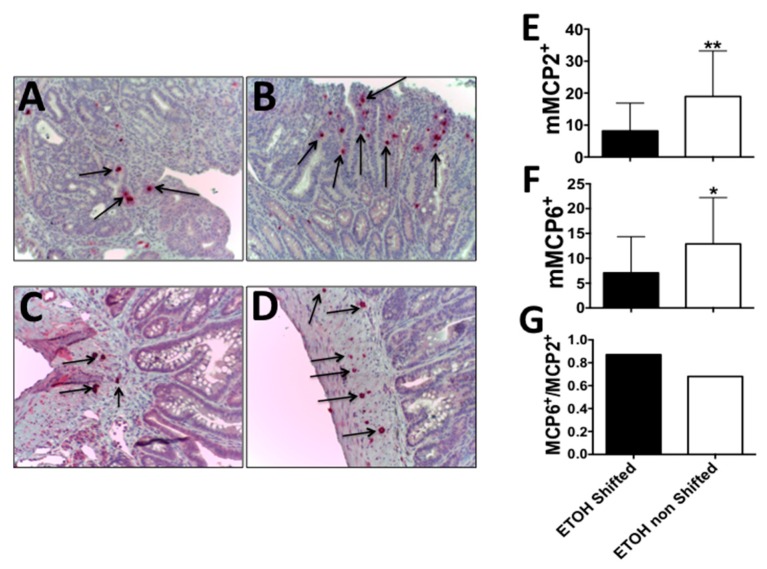
Accelerates tumorigenesis in shifted alcohol-treated mice is associated with a change in the mast cell phenotype towards MCP6^+^ cells: LD shift increases the ratio of stromal to intra-epithelial mast cells. Fixed and paraffin embedded polyps were stained for mMCP2 or mMCP6, and stained cells were counted using a light microscope. (**A**) Intraepithelial mMCP2^+^ mast cells in LD shifted + ethanol mice; (**B**) Same in ethanol non-shifted mice; (**C**) Stromal mMCP6^+^ mast cells in LD shifted + ethanol mice; (**D**) Same in ethanol non-shifted mice; Black arrows point to mast cells; (**E**) Mean values of mast cell counts in panels A + B; *n* = 24, *n* = 25 fields, respectively at 200× magnification, (** *p* = 0.003); (**F**) Mean values of mast cell counts in panels C + D; *n* = 28, *n* = 15 fields, respectively, at 200× magnification, (* *p* = 0.003); (**G**) Ratio of mMCP6 stromal to mMCP2 intraepithelial mast cells.

Our group (and others) have reported the important role of mast cells in polyposis [24,25,26,27]. We also showed that chronic alcohol consumption results in increased mast cell numbers at the polyp site [11]. Mast cells can be located intraepithelially (intestinal mucosa) and in the stroma (submucosa). Stromal mast cells express tryptase (mMCP6), and could play a role in promoting stromal activation and carcinogenesis in the colon [28]. Here, we stained the intestinal tissue for mMCP2 (chymase) and mMCP6 (tryptase) mast cells. Interestingly, while densities of intra-polyp mMCP2 and mMCP6 mast cells dropped with LD shift, the ratio of mMCP6 (stromal)/mMCP2 (intraepithelial) mast cells increased (0.87 relative to 0.68) (Figure 2). This finding is consistent with a pathogenic role of stromal localization of tryptase-positive mast cells in colon carcinogenesis [28].

### 2.3. Circadian Disruption Induces a Pro-Tumorigenic Dysbiosis in Alcohol-Fed Mice

**Figure 3:**
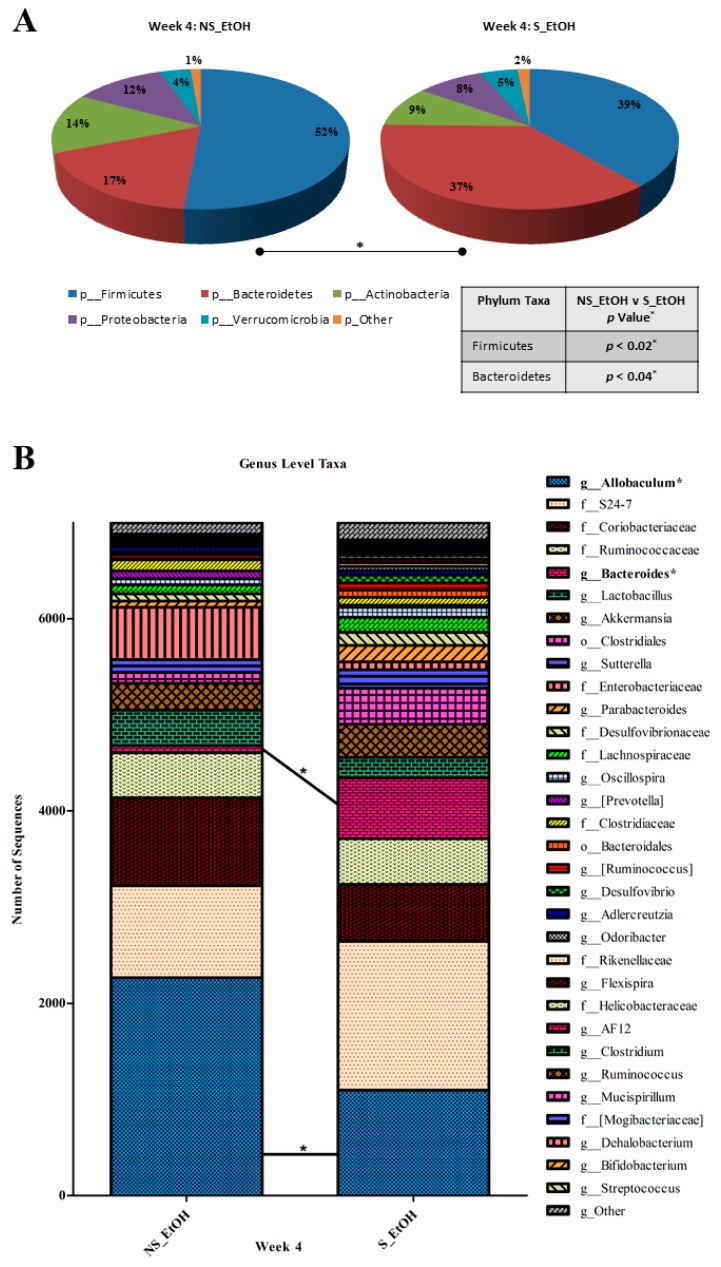
Light:dark (LD) shift changes microbiota in alcohol-fed mice. There were different relatively abundant phylum and genus microbial taxa in the fecal microbiomes of non-shifted, alcohol-fed mice (NS_EtOH) and LD shifted, alcohol-fed mice (S_EtOH) after four weeks of treatment. (**A**) The relative abundances of Bacteroidetes and Firmicutes, as well as (**B**) *Allobaculum* and *Bacteroides* are inversely proportional and different between NS_EtOH and S_EtOH mice feces. The average number of sequences was rarefied to 7000 sequences per sample. * denotes a significant difference between NS_EtOH and S_EtOH mice fecal samples.

The phenotype of mast cells can be altered by their surrounding environment, such as the gut microbiota [29]. Accumulating evidence suggests a role of altered microbiota and the resulting inflammation in colon carcinogenesis [30]; thus, it is possible that mast cells in alcohol-fed mice with circadian disruption could be a response to altered microbiota (e.g., dysbiosis). We therefore ran 16S rRNA gene-based analysis in these mice to study the potential role of microbiota in the interaction between alcohol consumption and circadian rhythm disruption in polyposis. The microbiota composition was similar between the two groups at baseline—before the start of experimental manipulations. Stool was collected and analyzed following four weeks of treatment. Treatment (alcohol and shifting) had a significant effect on the microbiota composition (Figure S1). In our treated animals, we observed significant differences in microbiota composition between alcohol-fed and shifted and non-shifted mice. Significant differences in α-diversity indices indicate that comparing groups have different microbiota composition [31,32]. There were significant differences in the Shannon diversity index (*p* = 0.02), Simpson’s diversity index (*p* = 0.04), Richness (*p* = 0.04), and Evenness (*p* = 0.04) between the alcohol-fed shifted mice and the control groups at the taxonomic level of genus (Table S1). The relative abundance of two phyla, three families, and two genera were affected by LD shift (*p* ˂ 0.05) (Table S2). At the taxonomic level of phylum, the relative abundance of Bacteroidetes was higher (*p* = 0.04), and Firmicutes (*p* = 0.02) was lower in the alcohol-fed shifted mice (Figure 3A, Table S2). Firmicutes and Bacteroidetes represent the two largest phyla in the mouse microbiota. The altered ratio of these phyla has been associated with a variety of inflammation-driven chronic pathologies, including metabolic syndrome and cancer [33,34]. Recently, in an adenomatous polyposis coli (APC)-based mouse model of polyposis, an altered Firmicutes/Bacteroidetes ratio was shown to be associated with altered tumor load [35]. Here we found that the ratio of Firmicutes/Bacteroidetes was significantly different between shifted and non-shifted groups (*p* = 0.02). Alcohol-fed shifted (experimental) mice showed a decrease in Firmicutes/Bacteroidetes ratio, which is reported to correlate with a decrease in short-chain fatty acid (SCFA) production, bacterial metabolites long known to have protective effects in colonic neoplastic transformation [36]. LD shifting in the alcohol-treated group altered the relative abundance of bacteria from the genus *Allobaculum* (phylum_Firmicutes; class_Erysipelotrichi) and the genus *Bacteroides* (phylum_Bacteroidetes; class_Bacteroidetes), with an observed decrease (*p* = 0.04) and increase (*p* = 0.04) compared to non-shifted mice, respectively (Figure 3B, Table S2).

## 3. Methods

### 3.1. Animals

Animal experiments were carried out at Northwestern University Feinberg School of Medicine, Chicago, IL, USA. The Institutional Animal Care and Use Committee of the Northwestern approved the animal protocol (protocol number: 2007-1284; the start date: 01/01/2008). Males and females show differences in circadian regulation and alcohol metabolism; therefore, only male mice were used.

We used TS4Cre × APC^lox468^ as our method of choice to model CRC. In this mouse model, polyposis is targeted to the terminal ileum and colon by utilizing epithelial expression of the fatty acid binding protein 1 (Fabp1). A Cre gene was inserted under the control of the Fabp1 gene promotor [37]. This mouse (known as Ts4cre) was then crossed to mice with LoxP flanking exons 11 and 12 of the adenomatous polyposis coli gene (APC^Δ468^). Double heterozygous mice for conditional APC^lox468^ and TS4-Cre therefore have conditional deletion in the APC protein that is restricted to the epithelial cells of the ileum and colon, deriving polyposis [38].

Age-matched four-week-old TS4Cre × APC^lox468^ mice were fed an alcohol (EtOH)-containing diet that is a modification of the Lieber DiCarli diet, where the fat calories are replaced by fish oil. Alcohol content was introduced to the diet at 3% and gradually increased to 15% over two weeks, followed by eight weeks for full amount of alcohol, as previously reported [11]. Therefore, the full concentration of alcohol was given when animal were >6 weeks of age. After two weeks of 15% alcohol, mice were randomly divided into two groups: LD shifting or maintained on a regular LD cycle for the duration of the experiment. The control group was maintained on a conventional 12 h light/12 h dark cycle (non-shifted), and the experimental group underwent a once weekly 12 h light/dark shift (i.e., a phase reversal of the light/dark cycle).

Weekly food consumption, caloric intake, and alcohol intake were not significantly different between the groups (data not shown). Mice were anesthetized and sacrificed between Zeitgeber time (ZT) ZT4 and ZT8. The intestinal tissue was divided into proximal and distal small intestine, and colon and tissues were examined by tissue microscope and then fixed in paraffin. Paraffin-embedded tissue was used for hematoxylin and eosin (H&E) and mast cell staining. Pictures of H&E stained slides were taken with an Olympus BX46 microscope, and were reviewed and quantified for adenomas by a gastrointestinal pathologist who was blind to treatment groups. SPSS version 23 (SPSS, Inc., Chicago, IL, USA) was used for all analyses. Proportions between categorical variables were compared between groups using the chi-square test or the Fisher′s exact test, where appropriate. numeric results (polyp size and numbers) are presented as mean ± S.E.M., and were compared using two-tailed ANOVA tests.

### 3.2. Tissue Staining and Immunohistochemistry

The paraffin blocks were cut in 5-lM sections. Polyclonal sheep anti-mouse mMCP2 antibody and rabbit anti-mouse mMCP6 were to stain for tryptase (mMCP6) and chymase (mMCP2), as previously described [11]. The MCP2- and MCP6-positive cells were quantified in the mucosal and submucosal parts of the polyps and compared between the groups. Statistical analysis was performed as stated above.

### 3.3. Microbial Community Structure Analysis

Total DNA was extracted from mice feces (FastDNA bead-beating Spin Kit for Soil, MP Biomedicals, Solon, OH, USA) collected at week 0 (baseline), and again after four weeks of experimental interventions. Primers (515F/806R) targeting the V4 variable region of microbial small subunit (SSU or 16S) ribosomal RNA (rRNA) genes were used for PCR [39], and prepared for next-generation sequencing using a modified two-step targeted amplicon sequencing (TAS) approach, as described previously [40]. Sequencing was performed using an Illumina MiSeq, with a V2 kit and paired-end 250 base reads at the University of Illinois at Chicago. Raw FASTQ files for each sample were processed using the software package PEAR (Paired-end read merger) (v0.9.8) [41]. The merged FASTQ files were imported into the software package CLC Genomics Workbench 8.0 (CLC Bio, Aarhus, Denmark, Qiagen, Venlo, The Netherlands). Primer sequences were removed, and sequences without both forward and reverse primers were discarded. Sequences were also trimmed using quality trimming algorithms (quality threshold, Q20) and length trimming (discarding everything less than 250 bp). The trimmed files were then exported as FASTA files into the software package QIIME (v1.8) [42] for chimera removal using the USEARCH6.1 algorithm [43]. The chimera-free FASTA files were then processed to cluster sequences into operational taxonomic units (OTUs) at a similarity threshold of 97% using the UCLUST algorism method. Representative sequences for each OTU were selected, and these sequences were annotated using the UCLUST and the Greengenes_13_8 reference (97_otus.fasta) and taxonomy database (97_otu_taxonomy.txt). These data were processed into a multi-taxonomic level biological observation matrix (BIOM; McDonald et al. 2012) [44]. The BIOM files were sub-sampled (rarefied) to the same number of sequences (7000 sequences/sample) to reduce the effect of variable library size on diversity measures [45]. Taxa with an average abundance of <1% across the entire sample set were removed from such analyses. Raw sequence data (FASTQ files) were deposited in the NCBI Sequence Read Archive. Differences in the relative abundance (RA) of individual taxa between different groups were tested using the “group_significance” algorithm, implemented within QIIME. Tests were done using the non-parametric Kruskal–Wallis one-way analysis of variance. To adjust for multiple comparisons, a false-discovery rate (FDR) adjusted *p*-value was calculated for each analysis. All data were exported to GraphPad Prism (v 5.03) software for Mann–Whitney *U* test for statistical differences between categorical variables, respectively. Statistical significance was set at *p*-value of ≤0.05.

## 4. Conclusions

In summary, we investigated an interaction between two habits commonly associated with the Western lifestyle—alcohol intake and circadian rhythm disruption—on CRC development. To model human CRC, we used TS4Cre × APC^lox468^ mice that develop polyps in the colon and distal ileum, unlike the APC *Min* mice that exhibit polyposis mainly within the small intestine. The invasive cancer developed only in the alcohol-fed, shifted mice, which had larger polyps and all developed advanced adenomas. Overall, our data suggests that LD shifting resulting in circadian rhythm disruption exacerbates alcohol-induced colon carcinogenesis and polyposis, and this “aggressive” phenotype change is associated with dysbiosis.

Emerging evidence has demonstrated that lifestyle-related factors such as obesity and metabolic syndrome are associated with low-grade inflammation, and these are also known to be risk factors for CRC [46,47,48]. It is well-established that alcohol—another established risk factor for CRC—causes gut inflammation. Our group has recently reported a link between gut inflammation and polyposis induced by alcohol in a mouse model of polyposis [11]. Therefore, it is plausible that the presence of another environmental factor that is pro-inflammatory could promote alcohol-induced colon carcinogenesis. We have previously shown that intestinal inflammation and pathologic effects of alcohol are exacerbated by shifting LD cycles in mice [13]. Here, we observed that LD shift accelerates alcohol-induced colon carcinogenesis, and is associated with a change in mast cell phenotype—mainly in the submucosal portion of the polyps. The mast cell shift from MCP2^+^ to MCP6^+^ suggests an inflammatory-mediated mechanism for the observed tumorigenesis in response to the combination of circadian rhythm disruption and alcohol consumption. These findings are consistent with a pro-tumorigenic role for mMCP6^+^ (tryptase+) mast cells [49], especially in the stroma where invasion occurs in CRC [28]. Our group (and others) previously showed that alcohol stimulates the expression of tryptase in mast cells [11,50]—an effect that is exacerbated by circadian disruption in our study—through as of yet unknown mechanisms. Several studies have shown the deleterious effect of circadian disruption (and particularly LD shifting) on the gut microbiota [51], and the microbiota could impact intestinal inflammation including mast cell phenotype, and consequently impact carcinogenesis [52]. Therefore, we analyzed and compared the microbiota of the shifted and non-shifted alcohol-fed mice as a possible mechanism for the accelerated inflammation and tumorigenesis. Changes in the microbiota as a result of the shift occurred as early as four weeks after treatment, preceding the polyposis in these mice that usually occurs after 8–10 weeks of age. Therefore, the microbiota changes are not the consequence of polyposis, and likely precede the tumorigenesis process. Thus, alterations in the microbiota resulting from LD shift could be a mechanism by which circadian disruption promotes alcohol-induced pro-tumorigenic inflammation and polyposis.

Our data shows that LD shifting exacerbates alcohol-induced colon carcinogenesis. The promotion of polyposis was associated with an elevated MCP6^+^/MCRP2^+^ ratio, suggestive of stromal activation—a pro-tumorigenic mechanism. Circadian disruption was associated with microbiota alteration in alcohol-fed mice. Together, our data shows that shifting exacerbates intestinal tumorigenesis in alcohol-treated mice by promoting a pro-tumorigenic inflammatory milieu, likely via changes in the microbiota. Further mechanistic studies are underway in our laboratory to explain this observation. These findings need to be confirmed with larger sample sizes, and to be explored in large-scale epidemiological studies.

## Supplementary Materials

Supplementary materials can be found at www.mdpi.com/1422-0067/17/12/2017/s1.
