# Supplementary material for: Light/Dark Shifting Promotes Alcohol-Induced Colon Carcinogenesis: Possible Role of Intestinal Inflammatory Milieu and Microbiota
Source: Int J Mol Sci. 2016 Dec 2;17(12):2017. doi: 10.3390/ijms17122017 (PMC5187817; doi:10.3390/ijms17122017)
Supplement: Supplementary file 1 [file ijms-17-02017-s001.pdf]

Supplementary Materials: Light/Dark Shifting Promotes Alcohol-Induced Colon Carcinogenesis: Possible Role of Intestinal Inflammatory Milieu and Microbiota

Faraz Bishehsari, Abdulrahman Saadalla, Khashayarsha Khazaie, Phillip A. Engen, Robin M. Voigt, Brandon B. Shetuni, Christopher Forsyth, Maliha Shaikh, Martha Hotz Vitaterna, Fred Turek and Ali Keshavarzian

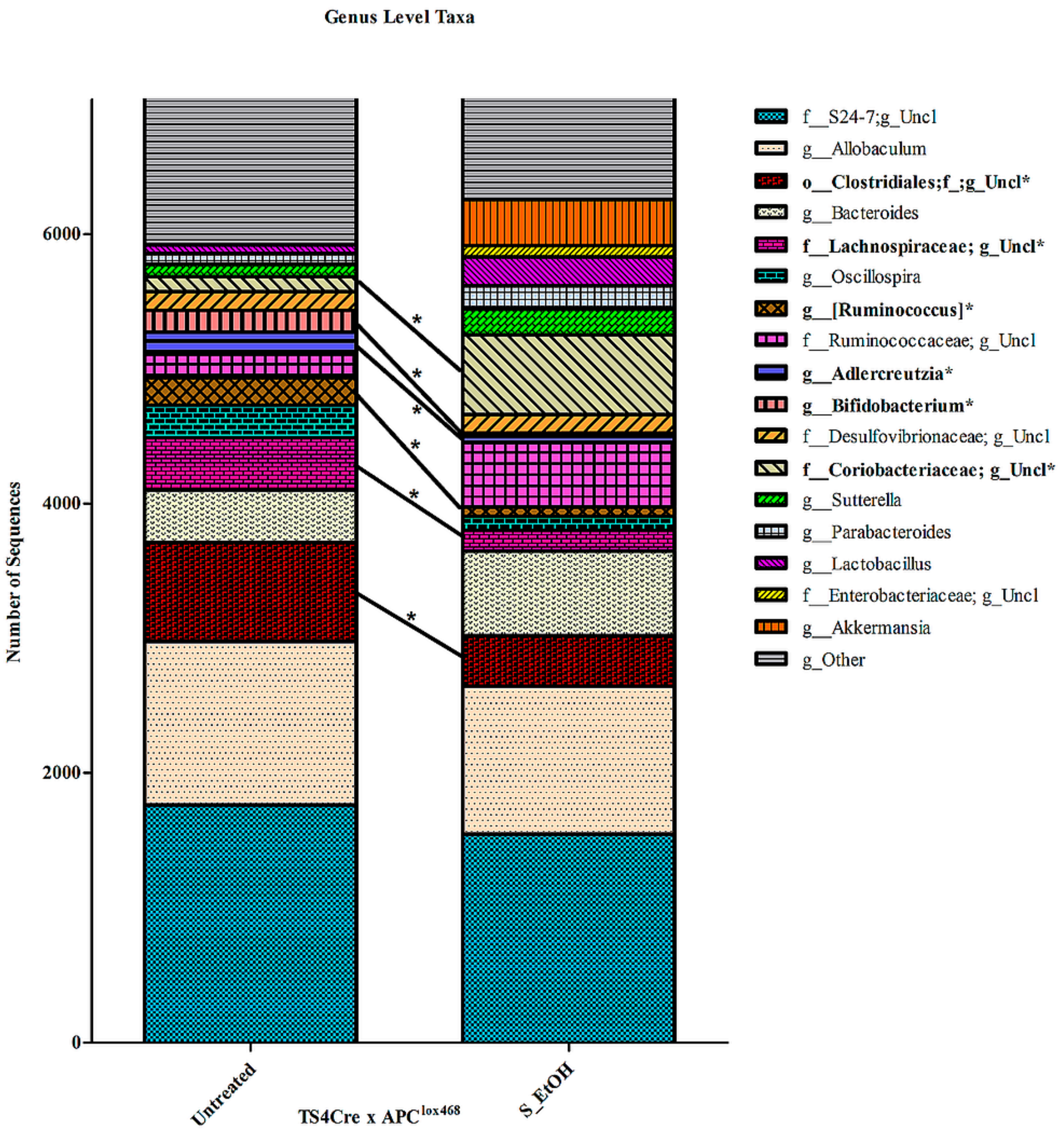

Figure S1. Effect of alcohol with Light:Dark (LD) shift on the microbiota. Genus taxa level relative abundances are inversely proportional and different between untreated and shifted alcohol (S_EtOH) TS4Cre × APC^{lox468} mice feces. The average number of sequences was rarefied to 7000 sequences per sample. * denotes taxa differences ($p < 0.05$) between untreated and S_EtOH mice fecal samples.

Table S1. α diversity indices.

Comparison	Taxonomic Level	α Diversity Index	*p*-Value [a]	Mean ± SEM	Mean ± SEM
		Feces: NS_EtOH vs. S_EtOH			
Feces: NS_EtOH vs. S_EtOH	Phylum	Species Richness (Margalef)	0.262 [a]	8.33 ± 0.33 (NS)	9.33 ± 0.49 (S)
Feces: NS_EtOH vs. S_EtOH	Phylum	Evenness (Pielou's)	0.905 [a]	0.61 ± 0.01 (NS)	0.60 ± 0.02 (S)
Feces: NS_EtOH vs. S_EtOH	Phylum	Shannon (log−e)	0.714 [a]	1.28 ± 0.05 (NS)	1.33 ± 0.06 (S)
Feces: NS_EtOH vs. S_EtOH	Phylum	Simpson (1−λ)	0.548 [a]	0.66 ± 0.01 (NS)	0.68 ± 0.02 (S)
Feces: NS_EtOH vs. S_EtOH	Class	Species Richness (Margalef)	0.095 [a]	14.67 ± 0.33 (NS)	16.33 ± 0.56 (S)
Feces: NS_EtOH vs. S_EtOH	Class	Evenness (Pielou's)	0.381 [a]	0.67 ± 0.14 (NS)	0.62 ± 0.02 (S)
Feces: NS_EtOH vs. S_EtOH	Class	Shannon (log−e)	0.905 [a]	1.81 ± 0.05 (NS)	1.74 ± 0.08 (S)
Feces: NS_EtOH vs. S_EtOH	Class	Simpson (1−λ)	0.262 [a]	0.80 ± 0.01 (NS)	0.76 ± 0.02 (S)
Feces: NS_EtOH vs. S_EtOH	Order	Species Richness (Margalef)	0.262 [a]	17.67 ± 0.88 (NS)	19.83 ± 0.98 (S)
Feces: NS_EtOH vs. S_EtOH	Order	Evenness (Pielou's)	0.548 [a]	0.63 ± 0.03 (NS)	0.59 ± 0.02 (S)
Feces: NS_EtOH vs. S_EtOH	Order	Shannon (log−e)	1.000 [a]	1.81 ± 0.05 (NS)	1.75 ± 0.08 (S)
Feces: NS_EtOH vs. S_EtOH	Order	Simpson (1−λ)	0.262 [a]	0.80 ± 0.01 (NS)	0.76 ± 0.02 (S)
Feces: NS_EtOH vs. S_EtOH	Family	Species Richness (Margalef)	0.548 [a]	35.00 ± 1.53 (NS)	36.17 ± 0.95 (S)
Feces: NS_EtOH vs. S_EtOH	Family	Evenness (Pielou's)	0.024 [a]	0.60 ± 0.01 (NS)	0.66 ± 0.01 (S)
Feces: NS_EtOH vs. S_EtOH	Family	Shannon (log−e)	0.024 [a]	2.12 ± 0.07 (NS)	2.35 ± 0.05 (S)
Feces: NS_EtOH vs. S_EtOH	Family	Simpson (1−λ)	0.095 [a]	0.82 ± 0.01 (NS)	0.85 ± 0.01 (S)
Feces: NS_EtOH vs. S_EtOH	Genus	Species Richness (Margalef)	0.048 [a]	47.33 ± 1.33 (NS)	52.67 ± 1.48 (S)
Feces: NS_EtOH vs. S_EtOH	Genus	Evenness (Pielou's)	0.048 [a]	0.57 ± 0.02 (NS)	0.63 ± 0.01 (S)
Feces: NS_EtOH vs. S_EtOH	Genus	Shannon (log−e)	0.024 [a]	2.21 ± 0.09 (NS)	2.50 ± 0.05 (S)
Feces: NS_EtOH vs. S_EtOH	Genus	Simpson (1-λ)	0.048 [a]	0.83 ± 0.01 (NS)	0.86 ± 0.01 (S)
Feces: NS_EtOH vs. S_EtOH	Species	Species Richness (Margalef)	0.167 [a]	51.33 ± 1.33 (NS)	57.67 ± 1.74 (S)
Feces: NS_EtOH vs. S_EtOH	Species	Evenness (Pielou's)	0.024 [a]	0.57 ± 0.02 (NS)	0.64 ± 0.01 (S)
Feces: NS_EtOH vs. S_EtOH	Species	Shannon (log−e)	0.024 [a]	2.24 ± 0.08 (NS)	2.57 ± 0.05 (S)
Feces: NS_EtOH vs. S_EtOH	Species	Simpson (1−λ)	0.024 [a]	0.83 ± 0.01 (NS)	0.87 ± 0.01 (S)

[a] $p < 0.05$, SEM = Standard Error Mean, NS_EtOH = non-shifted ethanol-fed mice, S_EtOH = shifted ethanol-fed mice; The groups were compared by the Mann–Whitney test.

Table S2. Relative abundance of sequences derived from mice fecal taxa.

Taxonomic Level	*p*-Value [a]	FDR-*p* [b]	S_ETOH # Seqs	NS_ETOH # Seqs
		Phylum		
Firmicutes	0.02 [a]	0.10	2738.33	3554.67
Bacteroidetes	0.04 [a]	0.10	2573.50	1277.67
Actinobacteria	0.12	0.20	655.00	990.67
Verrucomicrobia	0.52	0.65	332.00	271.33
Proteobacteria	1.00	1.00	596.00	810.00
		Family		
Rikenellaceae	0.02 [a]	0.21	74.83	15.00
Bacteroidaceae	0.04 [a]	0.21	621.00	82.67
Erysipelotrichaceae	0.04 [a]	0.21	1127.17	2261.67
Coriobacteriaceae	0.07	0.23	632.33	1004.00
Porphyromonadaceae	0.07	0.23	185.33	71.67
Desulfovibrionaceae	0.12	0.32	217.33	98.33
Lactobacillaceae	0.20	0.39	216.83	368.00
Clostridiales; Other	0.20	0.39	390.17	116.33
S24-7	0.30	0.54	1513.50	988.33
Helicobacteraceae	0.36	0.58	77.67	32.33
Lachnospiraceae	0.44	0.58	240.17	120.00
Alcaligenaceae	0.44	0.58	196.00	124.00
Enterobacteriaceae	0.52	0.64	87.17	549.67
Bifidobacteriaceae	0.56	0.64	23.83	0.67
Verrucomicrobiaceae	0.79	0.80	326.17	275.00
Ruminococcaceae	0.80	0.80	608.17	510.33
		Genus		
Bacteroides	0.04 [a]	0.33	632.67	73.67
Allobaculum	0.04 [a]	0.33	1107.67	2254.00
Parabacteroides	0.07	0.40	181.67	75.33
Adlercreutzia	0.20	0.47	50.50	56.67
Lactobacillus	0.30	0.47	219.17	353.33
Oscillospira	0.30	0.47	106.33	46.33
Sutterella	0.30	0.47	203.00	127.00
[Ruminococcus]	0.52	0.63	77.00	43.67
Akkermansia	0.79	0.90	329.33	288.67
Bifidobacterium	0.88	0.93	21.67	0.33

S_EtOH = shifted ethanol-fed mice; NS_EtOH = non-shifted ethanol-fed mice; # Seqs = average number of sequences in defined group; [a] *p*-value: $p < 0.05$; [b] False Discovery Rate: FDR-*p* < 0.05.
